# Sex‐specific heritabilities for length at maturity among Pacific salmonids and their consequences for evolution in response to artificial selection

**DOI:** 10.1111/eva.13579

**Published:** 2023-07-11

**Authors:** Madilyn M. Gamble, Ryan G. Calsbeek

**Affiliations:** ^1^ Graduate Program in Ecology, Evolution, Ecosystems, and Society Dartmouth College Hanover New Hampshire USA; ^2^ Department of Biological Sciences Dartmouth College Hanover New Hampshire USA

**Keywords:** artificial selection, heritability, intralocus sexual conflict, Pacific salmon

## Abstract

Artificial selection, whether intentional or coincidental, is a common result of conservation policies and natural resource management. To reduce unintended consequences of artificial selection, conservation practitioners must understand both artificial selection gradients on traits of interest and how those traits are correlated with others that may affect population growth and resilience. We investigate how artificial selection on male body size in Pacific salmon (*Oncorhynchus* spp.) may influence the evolution of female body size and female fitness. While salmon hatchery managers often assume that selection for large males will also produce large females, this may not be the case—in fact, because the fastest‐growing males mature earliest and at the smallest size, and because female age at maturity varies little, small males may produce larger females if the genetic architecture of growth rate is the same in both sexes. We explored this possibility by estimating sex‐specific heritability values of and natural and artificial selection gradients on length at maturity in four populations representing three species of Pacific salmon. We then used the multivariate breeder's equation to project how artificial selection against small males may affect the evolution of female length and fecundity. Our results indicate that the heritability of length at maturity is greater within than between the sexes and that sire–daughter heritability values are especially small. Salmon hatchery policies should consider these sex‐specific quantitative genetic parameters to avoid potential unintended consequences of artificial selection.

## 1 INTRODUCTION

Recent decades have seen increased recognition that the success of conservation and resource management efforts may improve if they consider evolutionary processes (Hendry et al., 2010; Mace & Purvis, 2008; Olivieri et al., 2016). This is true because human alteration of the environment coinciding with conservation policies is likely to alter selection pressures that may in turn affect evolution. For example, human‐induced artificial selection, whether intentional or inadvertent, may be a prevalent source of evolutionary change within conservation and management programs that involve domestication or captive breeding, where humans have a direct role in determining which organisms are allowed to pass on their genetic material (Conner, 2003). Still, these evolutionary impacts remain underappreciated in many conservation programs, a shortcoming that needs attention if we are to fully grasp the consequences of natural resource management policies.

Salmon hatcheries in the United States represent one type of breeding program in which artificial selection is common, often intentional, and yet relatively under‐studied in terms of its manifold evolutionary implications. Hatcheries spawn adult salmon, raise the eggs and juveniles in a controlled environment to increase egg and juvenile survival, and then release juveniles into the wild to continue their life cycle (Anderson et al., 2020). Most produce salmon primarily for harvest, though some have conservation goals. There is considerable variation in the degree to which hatchery and natural populations interbreed, meaning that selection in hatcheries may have effects on wild populations (Anderson et al., 2020). Because salmon life history traits such as body length and age at maturity are highly variable (Quinn, 2018) and have significant heritability values (Carlson & Seamons, 2008; Hankin et al., 1993; Hecht et al., 2015), artificial selection by hatcheries has ample opportunity to impact salmon evolution. Artificial selection on body size and migration timing has been observed and quantified in hatcheries (McLean et al., 2005), and this selection may have resulted in the evolution of return timing (Quinn et al., 2002; Tillotson et al., 2019). In light of these observations, policymakers have reviewed hatchery practices that may exert artificial selection in recent decades (Anderson et al., 2020; California HSRG, 2012). These reviews have often been motivated by evidence that hatchery‐origin salmon have reduced fitness compared with their natural‐origin counterparts in many species (Chinook (*Oncorhynchus tshawytscha*): Ford et al., 2012; Coho (*O. kisutch*): Thériault et al., 2011; steelhead (*O. mykiss*): Reisenbichler & McIntyre, 1977; Pink (*O. gorbuscha*): Shedd et al., 2022; Atlantic salmon (*Salmo salar*): Milot et al., 2012; see also Berejikian & Ford, 2004; Araki et al., 2008).

A common hatchery practice that has come under scrutiny is the exclusion of individuals exhibiting certain life history tactics, particularly those tactics resulting in small, early‐maturing individuals such as “jacks” and mature male parr, from the broodstock (Anderson et al., 2020; California HSRG, 2012; Mobrand et al., 2004; Myers & Hutchings, 1986). Jacks are characterized as anadromous males that mature at least 1 year earlier than the youngest females, whereas mature male parr sexually mature and reproduce without ever migrating to the ocean. Variation in size among these male age classes is correlated with alternative reproductive tactics. Large anadromous “hooknose” males develop larger protruding jaws and sharper teeth that help them compete aggressively for access to females preparing nest sites; jacks and mature male parr are more morphologically similar to females or juvenile salmon, respectively, and avoid fighting by sneaking copulations (Berejikian et al., 2010). Age at maturity in salmonids is believed to be determined by an underlying threshold related to body size, such that the fastest‐growing individuals mature earliest (Hutchings & Jones, 1998; Hutchings & Myers, 1994); therefore, jacks represent the fastest‐growing males. The frequency of these alternative tactics varies among salmon species and populations (Myers et al., 1986; Quinn, 2018). The management decision to exclude these sneaker‐male tactics from hatchery broodstocks is based on the goal of producing large, anadromous salmon for commercial harvest and recreational fishing, the high overall heritability values of age and size at maturity, and the assumption that age and size at maturity have high intersexual heritability values so selection will act similarly between the sexes (California HSRG, 2012). Hatchery policies regarding the degree to which jacks and mature male parr should be included in broodstocks vary among states, salmon species, and hatcheries (California HSRG, 2012; Mobrand et al., 2004). Some hatchery managers have begun to recommend including these younger, smaller males in broodstock (Mobrand et al., 2004) because the greater intraspecific variation afforded by alternative life histories lends increased population resilience to salmon stocks (Moore et al., 2014).

Although the maintenance of population‐level genetic diversity and resilience to ecological stochasticity is one important consideration, there is evidence, both theoretical (Gamble & Calsbeek, 2023) and empirical (Bielak et al., 2014; Sinervo & Zamudio, 2001), suggesting that small bodied males employing satellite and sneaker mating tactics may produce daughters with larger bodies and higher fitness. This counter‐intuitive pattern arises because, although males and females share most of the same genes, the attributes of high‐fitness males often differ from the attributes of high‐fitness females (Cox & Calsbeek, 2009), resulting in sexually antagonistic selection and potentially intralocus sexual conflict (Bonduriansky & Chenoweth, 2009; Rice & Chippindale, 2001). To resolve the potential genomic conflicts that arise from this axiom of sexually reproducing species, the genetic correlations for fitness‐related traits like body size are often found to be zero or negative when measured between parents and their opposite‐sex progeny (Cox & Calsbeek, 2009; Foerster et al., 2007). To our knowledge, conservation biologists and captive breeding program managers have yet to investigate how this phenomenon, well‐documented in other species, may apply to salmonids. If salmonids follow a similar pattern, management strategies and policies may need to be adjusted accordingly.

This oversight is problematic for salmon conservation practitioners and managers because female age and size at maturity affect fecundity (Allen, 1958; Beacham, 1982; Beacham & Murray, 1993; Ohlberger et al., 2023) and subsequent population growth rates, which are key metrics for hatcheries attempting to increase salmon production or conserve threatened populations. If male and female length at maturity are genetically correlated, selection on males may affect evolution in females (Falconer, 1989). Indeed, the fastest‐growing males mature earliest (Hutchings & Myers, 1994; Vøllestad et al., 2004), growth rate is significantly heritable in salmonids (*h* ^*2*^ = 0.16–0.46; Gjedrem, 2000; Hecht et al., 2015; Kristjánsson et al., 2020; Silverstein & Hershberger, 1995), and because females vary less than males in age at maturity in most salmon species (Fleming, 1998; Quinn, 2018), small, slow‐growing, early‐maturing males may produce larger, more fecund daughters. If true, excluding the youngest and smallest mature males from hatchery broodstocks may inadvertently hinder hatchery goals of producing more and larger salmon. Understanding the evolutionary implications of artificial selection against small male salmon requires measuring the heritability values of length at maturity both within *and* between the sexes. Furthermore, because the genetic architecture underlying age and size at maturity, and the sex specificity of this genetic architecture, varies among salmon species and populations (Waters et al., 2021), heritability values should be estimated separately for the various salmon populations and species that are subjected to selection in hatcheries.

Here, we take advantage of existing pedigreed datasets for four populations representing three species of Pacific salmonids to understand how artificial selection on male length at maturity could drive a correlated evolutionary response in females. Specifically, our objectives for each population were (1) to estimate sex‐specific heritability values for length at maturity, (2) quantify natural and artificial selection on male and female length at maturity, and (3) use heritability estimates and selection gradients to project how selection on male length will affect the evolutionary response in females. Our study provides a first step toward understanding how the growing literature on intralocus sexual conflict can inform better management decisions in the conservation of an ecologically and economically important fish species.

## 2 METHODS

### 2.1 Datasets

We quantified sex‐specific heritability estimates of length at maturity using four datasets from Chinook salmon (*O. tchawytscha*), Coho salmon (*O. kisutch*), and steelhead (*O. mykiss*) populations. Phenotypic data and tissue samples for genetic parentage assignment were collected as adults migrated upstream to spawn. All datasets included pedigree data (identification of dam, sire, and offspring), sex, length at maturity, origin (wild origin, natural origin, or hatchery origin; see below). None of these datasets included offspring without known parentage. A summary of the datasets used is given in Table 1.

**TABLE 1:** Summary statistics for datasets used, including brood years, range of lengths for both sexes, the number of sires and dams, the mean number of offspring for sires and dams, and the number of offspring of both sexes.

Brood year	Mean length ± SD (mm) (females/males)	Sires		Dams		Offspring
		*N*	Mean# offspring	*N*	Mean# offspring	*N* (daughters/sons)
Chinook (Wenatchee River, WA)						
2004	808.92 ± 93.75 (813.64 ± 60.63/804.22 ± 117.71)	105	1.9	122	1.7	203 (106/97)
Coho (Cedar River, WA)						
2005	641.96 ± 104.69 (652.04 ± 75.80/632.74 ± 124.85)	51	4.4	45	5.0	227 (124/103)
Coho (Umpqua River, OR)						
2002	709.47 ± 104.12 (735.56 ± 47.17/683.84 ± 134.17)	200	2.6	194	2.6	512 (255/257)
2003	696.18 ± 114.97 (728.22 ± 49.24/667.60 ± 145.44)	122	2.2	107	2.5	263 (125/138)
Steelhead (Snow Creek, WA)						
1982–2000	646.34 ± 65.56 (650.52 ± 56.62/641.45 ± 74.43)	84	2.0	141	2.4	811 (437/374)

The Chinook dataset was collected between 2004 and 2009 from a spring‐run, hatchery‐supplemented population from the Wenatchee River, Washington, USA (Ford et al., 2012). Briefly, all fish sampled were genotyped at 11 microsatellite loci with an estimated genotyping error rate of about 1%. Parentage assignment was conducted in the program FAMOZ (Gerber et al., 2003) using the likelihood method developed by (Meagher & Thompson, 1986). In this dataset, 72.6% of offspring were assigned to two parents. For offspring‐assigned parents, the mean posterior probability of correct assignment was 95.2%. For a complete explanation of genotyping and parentage analysis methods for this dataset, see Ford et al. (2012), Williamson et al. (2010), and Ford and Williamson (2010).

One of the Coho salmon datasets was collected between 2003 and 2008 from a population re‐colonizing upper reaches of the Cedar River, Washington, USA following a dam removal (Anderson et al., 2010). All individuals were genotyped at 10 microsatellite markers with an estimated error rate of 0.66%. Parentage analysis was conducted through the program Cervus version 3, which employs a likelihood ratio method (Kalinowski et al., 2007; Marshall et al., 1998). Parentage assignment was estimated to be correct in 97.7% of cases. However, the proportion of offspring assigned to at least one parent was low: 20.1%, 44.7%, 74.5%, and 71.1% for return years 2005, 2006, 2007, and 2008, respectively. Full details of the genotyping and parentage analysis methods can be found in Anderson et al. (2010).

The other Coho salmon dataset was collected between 2002 and 2006 from a hatchery‐supplemented population in the Umpqua River, Oregon, USA (Banks et al., 2013; Thériault et al., 2011). Individuals were genotyped at 10 microsatellite loci; error rate was not reported by the authors. Parentage analysis was conducted using the program PASOS version 1.0, which uses a maximum likelihood approach followed by exclusion to assign offspring to parents (Duchesne et al., 2005). The majority of offspring were assigned to one or both parents in all years (76%, 90%, 94%, and 97% for brood years 2003, 2004, 2005, and 2006, respectively). Simulations suggested that parentage assignment was correct for 91%, 96%, 96%, and 97% of offspring in brood years 2003, 2004, 2005, and 2006, respectively. Full genotyping and parentage analysis details can be found in Thériault et al. (2011).

The steelhead dataset was collected between 1986 and 2004 from a wild population in Snow Creek, Washington, USA (Seamons et al., 2007). All individuals were genotyped at 12 microsatellite loci with an error rate of 0.6%. Parentage analysis was conducted with the program WHICHPARENTS 1.0, which matches offspring with parents that share at least one allele at all loci. Across all years, 65.7% of adult offspring were successfully assigned to parents; this number varied from 48% to 95% (mean = 73%) across years. This study did not estimate the correctness rate of parentage assignment. Full details can be found in Seamons et al. (2004, 2007).

We used only wild‐origin (those with little to no hatchery ancestry) or natural‐origin fish (those that may have hatchery ancestry but were reared in the wild) in each dataset to reduce the environmental effect of hatchery rearing on growth rate and maturation schedule (Larsen et al., 2004, 2006, 2013; McKinney et al., 2020) and understand how natural selection operates on length at maturity outside hatcheries. In all cases, wild‐ or natural‐origin fish were identified as those with an adipose fin, since hatcheries clip the adipose fins of fish they release. For the Chinook dataset, natural‐origin fish were defined as those whose parents spawned in the wild; individuals may still have had ancestors spawned in a hatchery (Ford et al., 2012). For the Umpqua Coho dataset, natural‐origin fish were those whose parents spawned in the wild or whose parents were spawned in a hatchery but who were released as unfed fry (Thériault et al., 2011). This unfed fry, though products of hatchery breeding, would not have experienced the augmented juvenile growth environment typical of hatcheries, so still fit our criteria for “wild” fish. Unfed fry was estimated to make up about 15% of adult returns in the Umpqua Coho dataset (Thériault et al., 2011). For the Cedar River Coho dataset, natural‐origin fish were identified as those with an adipose fin; the origin of their parents was not considered (Anderson & Quinn, 2007). The steelhead dataset was collected from a wild‐origin population such that any natural‐origin or hatchery‐origin fish in the original dataset were strays from other populations (Seamons et al., 2007); these were removed prior to analysis. For simplicity, we hereafter refer to all fish and populations as “natural‐origin.”

### 2.2 Sex‐specific heritabilities

To facilitate comparisons among populations and species, we first standardized length at maturity within each population, within each year (spawn year for parents and brood year for offspring), and for each sex in units of standard deviation. We did this in each case, by subtracting the population‐, year‐, and sex‐specific mean length from each value and dividing by the standard deviation. For the steelhead population, some individuals were iteroparous and thus had offspring from multiple brood years. In these cases, we calculated standardized length separately for each year in which the individual spawned.

Following preliminary analyses suggesting the potential for different heritability values between the sexes (see Table S1), we estimated sex‐specific heritabilities as twice the slope from the regression of sex‐specific mean standardized offspring length against the standardized length of each sire and dam in each population and year (*h* ^*2*^ = 2*b*_*op*_; Falconer, 1989). This approach has been used to estimate heritability values in other wild populations, including populations of salmonids (Abadía‐Cardoso et al., 2013; Dickerson et al., 2005; Gamble & Calsbeek, 2021).

We tested whether each single parent–offspring regression slope differed significantly from zero (*h* ^*2*^ ≠ 0). For iteroparous steelhead, we treated offspring from different brood years as separate families and calculated mean offspring lengths separately for each brood year. We conducted these analyses separately for each year in which there were >30 family means for each sex‐specific parent–offspring combination. Because none of the years in the Steelhead dataset satisfied this minimum sample size, and because narrow‐sense heritabilities can only be estimated between consecutive generations, we instead estimated the slope of the parent–offspring regression, as this relationship may still reveal the degree to which offspring resemble their parents due to shared genes. We did not specifically investigate maternal effects in these models, both because of small sample sizes and because maternal effects on length in salmonids tend to diminish within the first few months after hatching (Forest et al., 2016; Heath et al., 1999; Silverstein & Hershberger, 1992, 1994). Because sample sizes were small, we ran a bootstrap analysis with 1000 replicates for each parent–offspring regression and calculated the bootstrapped mean and 95% confidence intervals for the slope of the regression. These were then doubled to generate bootstrapped mean and 95% confidence intervals of heritability estimates (Table S2). This analysis was performed using the *boot* package in R. All analyses were run in R (R Core Team, 2020).

### 2.3 Selection analysis

We conducted two different selection analyses for each Chinook and Coho population and year: one to estimate the strength and form of natural selection experienced in the wild, and a second to approximate an artificial selection regime similar to that experienced in hatcheries that exclude jacks from the broodstock. The steelhead population was excluded from these selection analyses because selection estimates for this population have already been published (Seamons et al., 2007). We estimated sex‐specific linear and quadratic natural selection gradients for each population and year by regressing relative reproductive success on standardized length and the square of standardized length (Arnold & Wade, 1984b; Lande & Arnold, 1983). We defined reproductive success for each individual as the number of offspring that returned to spawn.

We estimated linear selection as the regression coefficient (β_1_ ± 1 SE) of relative reproductive success (reproductive success divided by its mean) on standardized length from a model with no additional terms (Brodie III et al., 1995; Lande & Arnold, 1983):

$$w=\beta_{0}+\beta_{1}\text{StdLength}+\varepsilon$$

We estimated quadratic selection using the partial regression coefficient of relative reproductive success on the square of standardized length from a model that also included the linear term:

$$w=\beta_{0}+\beta_{1}\text{StdLength}+\gamma {\text{StdLength}}^{2}+\varepsilon$$

Quadratic selection gradients were obtained by doubling the quadratic regression coefficient and its standard error (*γ* ± 1 SE; Phillips & Arnold, 1989). Statistical significance of linear and quadratic selection gradients was assessed using generalized linear models with a Poisson distribution and a logit link function relating absolute fitness to standardized length (Arnold & Wade, 1984a; Lande & Arnold, 1983). A Poisson distribution was used because absolute reproductive success values were all positive integers. We compared the 95% CIs of selection gradients between males and females within each population to determine if they were statistically different. Selection analyses were performed in R v. 4.0.3 (R Core Team, 2020).

We used these same datasets to simulate an artificial selection regime on male length at maturity in which the younger and smaller males (jacks) are not allowed to reproduce at all. Jacks are usually defined as males that mature at least 1 year younger than the youngest females (Berejikian et al., 2010; Quinn, 2018). This method was used for the Chinook population, as age data were available. For the Umpqua Coho population, the original dataset included a determination of male life history based on a bimodal length distribution (Thériault et al., 2011); we retained the original life history determination in our analyses. For the Cedar River Coho population, we used a bimodal male length distribution to identify jacks (Figure S1). To estimate hypothetical artificial selection gradients on male length at maturity (β_m_) for each population and year in which heritability estimates could be calculated, we assigned every male a “fitness” of 0 or 1 depending on whether that individual was a jack or not, respectively. Assigning jacks a fitness of 0 represents their complete exclusion from hatchery broodstock, while assigning hooknose males a fitness of 1 (rather than their observed fitness) reflects both inflated fitness because of the exclusion of jacks and the lack of sexual selection occurring in a hatchery environment. The distinction between jacks and hooknose males was made using age data, a length cutoff determined by bimodal length distributions, or using the life history strategy assigned to each male in the original dataset, depending on the data available for each population. We then regressed relative fitness (0 or 1 divided by the mean) on standardized length and the square of standardized length as described above to arrive at hypothetical linear and quadratic artificial selection gradients (Arnold & Wade, 1984b; Lande & Arnold, 1983). Owing to the lack of within group variance in reproductive success (e.g., hypothetical reproductive success was perfectly predicted by length), we report the significance of these selection gradients from nonparametric regression using rank‐based estimation implemented through the *rfit* function in the R package *Rfit* (R Core Team, 2020). All analyses were run in R (R Core Team, 2020).

### 2.4 Breeder's equation projections

The multivariate breeder's equation (Δ**z** = **Gβ**) predicts evolutionary change in the mean phenotype arising due to selection on heritable traits (Lande, 1979; Lande & Arnold, 1983). In this equation, **β** is a vector of partial regression coefficients representing the total direct selection acting on each trait, **G** is a matrix of additive genetic variances (*V*_*a*_) and covariances (*Cov*_*a*_) among traits, and Δ**z** is a vector of estimated changes in mean phenotypes. Because heritability is the quotient of additive genetic variance and phenotypic variance, and because using standardized length at maturity results in a phenotypic variance of 1 for each population, sex, and year, we used intrasexual heritability estimates as sex‐specific additive genetic variances for standardized length at maturity. Likewise, intersexual heritability estimates served as additive genetic covariances for standardized length at maturity between males and females.

We used two sets of selection gradients in our breeder's equation projections and compared their evolutionary outcomes in units of standard deviations of length. The first set was comprised of the sex‐specific linear and quadratic selection gradients estimated from regressions of relative reproductive success on standardized length as described above, representing natural selection acting in the wild. The second set of selection gradients was the hypothetical set representing artificial selection on length at maturity via the exclusion of jacks from hatchery broodstock. For this second set of selection gradients, we held selection on female length at maturity (β_f_) at the value estimated under natural selection so we could compare the how natural and artificial selection on male length affects female evolution. We quantified uncertainty in our projections of evolution by populating the breeder's equation with means, means +1 SE, and means – 1SE for all parameters.

Finally, we evaluated the effect of changing female length on fecundity. There are many published estimates of the relationship between length and fecundity for Pacific salmon, and these relationships change with latitude and freshwater migration distance (Beacham & Murray, 1993). Using a published estimate of the linear fecundity–length relationship for Coho salmon in Puget Sound, WA (Allen, 1958; Beacham, 1982), which have a similar latitude and migration distance to the Coho populations investigated here, we translated projected length changes into projected changes in fecundity for each Coho population and year by multiplying the projected change in length by 14.05 eggs per mm (Beacham, 1982).

## 3 RESULTS

### 3.1 Datasets

When broken down into sex‐specific single‐parent–offspring families, there were four population‐year combinations in which all four sets of single‐sex parent–offspring sample sizes were greater than 30 families (Chinook: 2004; Cedar River Coho: 2005; Umpqua River Coho: 2002, 2003). Because none of the steelhead brood years had adequate sample sizes, we report an analysis pooled across years for the steelhead population alongside the yearly analyses from other populations. Results from all other pooled years analyses are reported in the Appendix S1.

### 3.2 Sex‐specific heritabilities

**FIGURE 1:**
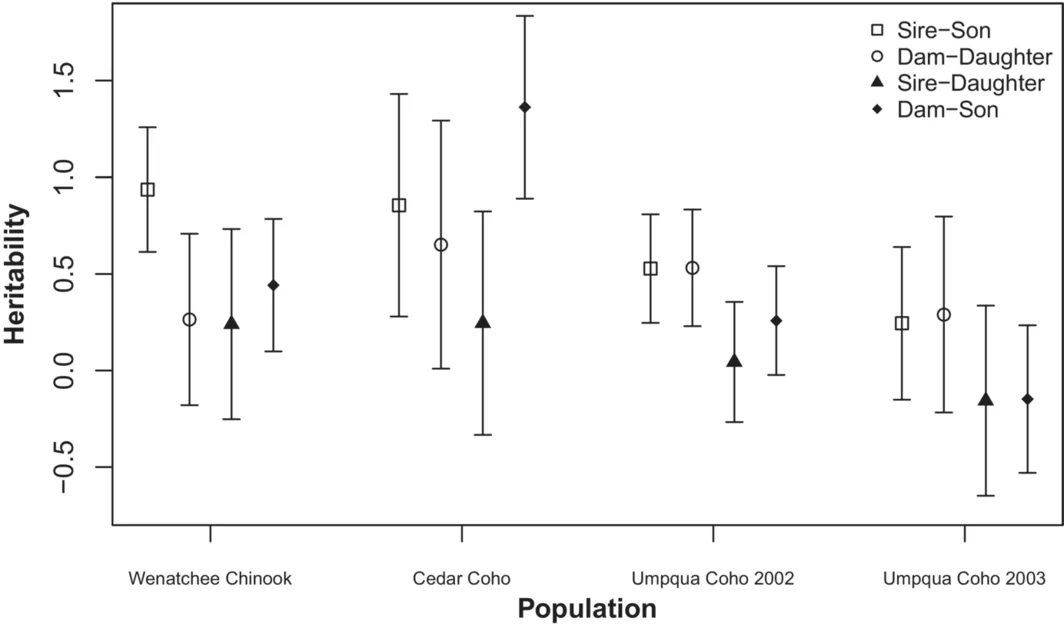
Sex‐specific heritabilities across populations and years.

The strength and direction of relationships between parent and offspring lengths differed among populations and sexes (Figure 1, Table S2). In Wenatchee River Chinook, all four sex‐specific heritability estimates were positive, though the dam–daughter and sire–daughter heritability estimates were not significantly different from zero (Figure S2). Sire–son heritability (mean ± 95% CI *h* ^*2*^ = 0.936 ± 0.322) had the largest magnitude, followed by the dam‐son heritability (*h* ^*2*^ = 0.441 ± 0.343); the dam–daughter (*h* ^*2*^ = 0.264 ± 0.443) and sire–daughter (*h* ^*2*^ = 0.240 ± 0.492) heritability estimates were the smallest. The Cedar River Coho population also exhibited positive heritability estimates in all four sex‐specific parent–offspring relationships, though the sire–daughter heritability estimate did not differ significantly from zero and the daughter–dam heritability estimate was only marginally significantly greater than zero (Figure S3). The dam–son (*h* ^*2*^ = 1.362 ± 0.473) and sire–son (*h* ^*2*^ = 0.855 ± 0.576) heritability estimates were greatest in magnitude, followed by dam–daughter heritability (*h* ^*2*^ = 0.651 ± 0.642); the sire–daughter heritability estimate (*h* ^*2*^ = 0.245 ± 0.578) was smallest. The Umpqua River Coho population exhibited similar trends in both 2002 and 2003 (Figure S4): the two intrasexual heritability estimates were positive and similar to each other in magnitude (2002: sire–son *h* ^*2*^ = 0.527 ± 0.281, dam‐daughter *h* ^*2*^ = 0.531 ± 0.302; 2003: sire–son *h* ^*2*^ = 0.244 ± 0.395, dam–daughter *h* ^*2*^ = 0.289 ± 0.507), though they were significantly greater than zero only in 2002. Both intersexual heritability estimates were small in magnitude (2002: sire–daughter *h* ^*2*^ = 0.044 ± 0.311, dam–son *h* ^*2*^ = 0.258 ± 0.282; 2003: sire–daughter *h* ^*2*^ = −0.156 ± 0.492, dam–son *h* ^*2*^ = −0.148 ± 0.382) and not significantly different from zero in either year. The Snow Creek steelhead population also exhibited positive and significant intrasexual parent–offspring regressions of similar magnitude (sire–son mean ± SE β = 0.294 ± 0.121, dam–daughter β = 0.232 ± 0.072; Figure S5). The dam–son regression (β = −0.181 ± 0.081) had a small, negative slope that differed significantly from zero, while the sire–daughter regression had a nonsignificant, near‐zero slope (β = −0.008 ± 0.127). Results of the bootstrap analyses supported the results of each significance test in every population (Table S2).

### 3.3 Selection analysis

#### 3.3.1 Natural selection

Linear natural selection gradients on length were positive and significantly greater than zero in all populations and sexes except for female Chinook (Table 2). Linear natural selection gradients were significantly greater in males than in females in the Umpqua River Coho population in both 2002 (mean ± 95% CI β_female_ = 0.136 ± 0.093, β_male_ = 0.299 ± 0.100) and 2003 (β_female_ = 0.124 ± 0.143, β_male_ = 0.352 ± 0.158). In the Chinook population, the linear natural selection gradient on male length (β = 0.136 ± 0.137) was also greater than that on female length (β = 0.067 ± 0.912), though not significantly so. In the Cedar River Coho population, the linear natural selection gradient on females (β = 0.458 ± 0.240) was greater than that on males (β = 0.302 ± 0.232), though this difference was not significant.

**TABLE 2:** Sex‐specific linear (β) and quadratic (*γ*) natural selection gradients on standardized length at maturity and their statistical significance for each population and year.

Sex	β ± 95% CI	*χ* ^2^ (*df*, *N*)	*p*	*γ* ± 95% CI	*χ* ^2^ (*df*, *N*)	*p*
Chinook (Wenatchee River, WA) – 2004						
Female	0.067 ± 0.912	1.563 (1122)	0.2	0.002 ± 0.139	0.002 (1122)	1.0
Male	**0.136 ± 0.137**	5.095 (1105)	0.02	0.031 ± 0.166	0.033 (1105)	0.9
Coho (Cedar River, WA) – 2005						
Female	**0.458 ± 0.240**	42.619 (1,55)	<0.0001	**−0.120 ± 0.396**	10.083 (1,55)	0.001
Male	**0.302 ± 0.232**	26.136 (1,70)	<0.0001	0.112 ± 0.477	0.000 (1,70)	1.0
Coho (Umpqua River, OR) – 2002						
Female	**0.136 ± 0.093**	23.628 (1449)	<0.0001	0.017 ± 0.121	0.008 (1449)	0.9
Male	**0.299 ± 0.100**	132.850 (1457)	<0.0001	**0.256 ± 0.186**	8.974 (1457)	0.003
Coho (Umpqua River, OR) – 2003						
Female	**0.124 ± 0.143**	7.810 (1232)	0.005	**−0.151 ± 0.176**	12.742 (1232)	0.0004
Male	**0.352 ± 0.158**	82.291 (1260)	<0.0001	0.098 ± 0.394	0.164 (1260)	0.7

*Note*: Selection gradients are presented with 95% confidence intervals; bold font indicates statistical difference from zero. Chi‐square test statistics (*χ* ^2^) are presented with degrees of freedom (*df*) and sample size (*N*).

Most quadratic natural selection gradients were small in magnitude and did not differ significantly from zero (Table 2). Quadratic natural selection differed significantly between the sexes in the Cedar River Coho population such that females experienced significant negative quadratic selection (*γ* = −0.120 ± 0.396) while males did not experience significant quadratic selection (*γ* = 0.112 ± 0.477). Quadratic selection gradients also differed between the sexes in the Umpqua River Coho population in 2002 such that males experienced significant positive quadratic selection (*γ* = 0.256 ± 0.186) while females did not experience significant quadratic selection (*γ* = 0.017 ± 0.121). There were no significant differences in quadratic selection gradients between the sexes in the Chinook population (*γ*_female_ = 0.002 ± 0.139, *γ*_male_ = 0.031 ± 0.166) or the Umpqua River Coho population in 2003 (*γ*_female_ = −0.151 ± 0.176, *γ*_male_ = 0.098 ± 0.394).

#### 3.3.2 Artificial selection

For the Chinook population in 2004, all females returned at age 4, therefore jacks were identified as males returning at age 3. Only one male was identified as a jack in this population; we thus did not conduct an artificial selection analysis nor breeder's equation projections using artificial selection gradients given the lack of variance in hypothetical fitness in males (i.e., all but one male had a fitness value of 1).

Age data were not available for all individuals in the Cedar River Coho population, so jacks were identified as males shorter than 500 mm length based on a bimodal length distribution (females exhibited a unimodal length distribution). 28 males were identified as jacks. Artificial selection against these males resulted in positive directional selection (mean ± 95% CI β = 0.372 ± 0.178; *t* = 5.85, *p* < 0.0001, *N* = 70; Table 3) and negative quadratic selection (*γ* = −0.217 ± 0.321; *t* = −6.31, *p* < 0.0001, *N* = 70) on male length at maturity.

**TABLE 3:** Sex‐specific linear (β) and quadratic (*γ*) artificial selection gradients on standardized male length at maturity and their statistical significance for each population and year.

β ± 95% CI	*t* (*N*)	*p*	*γ* ± 95% CI	*t* (*N*)	*p*
Coho (Cedar River, WA) – 2005					
**0.372 ± 0.178**	5.85 (70)	<0.0001	**−0.217 ± 0.321**	−6.31 (70)	<0.0001
Coho (Umpqua River, OR) – 2002					
**0.378 ± 0.020**	67.31 (457)	<0.0001	**−0.146 ± 0.014**	−108.22 (475)	<0.0001
Coho (Umpqua River, OR) – 2003					
**0.490 ± 0.030**	71.81 (260)	<0.0001	**−0.497 ± 0.020**	−63.81 (260)	<0.0001

*Note*: Selection gradients are presented with 95% confidence intervals; bold font indicates statistical difference from zero. Nonparametric regression test statistics (*t*) are presented with sample size (*N*).

The Umpqua River Coho dataset identified jacks as males shorter than 500 mm based on a bimodal length distribution (Thériault et al., 2011); we retained the original classification for this analysis. In 2002, 73 jacks were identified in this population; excluding them from reproductive success resulted in a positive directional selection gradient (β = 0.378 ± 0.020; *t* = 67.31, *p* < 0.0001, *N* = 457) and a negative quadratic selection gradient (*γ* = −0.146 ± 0.014; *t* = −108.22, *p* < 0.0001, *N* = 457) on male length at maturity. In 2003, 59 jacks were identified in this population; excluding them from reproductive success resulted in a positive directional selection gradient (β = 0.490 ± 0.030; *t* = 71.81, *p* < 0.0001, *N* = 260) and a negative quadratic selection gradient (*γ* = −0.497 ± 0.020; *t* = −63.81, *p* < 0.0001, *N* = 260) on male length at maturity.

### 3.4 Breeder's equation projections

**FIGURE 2:**
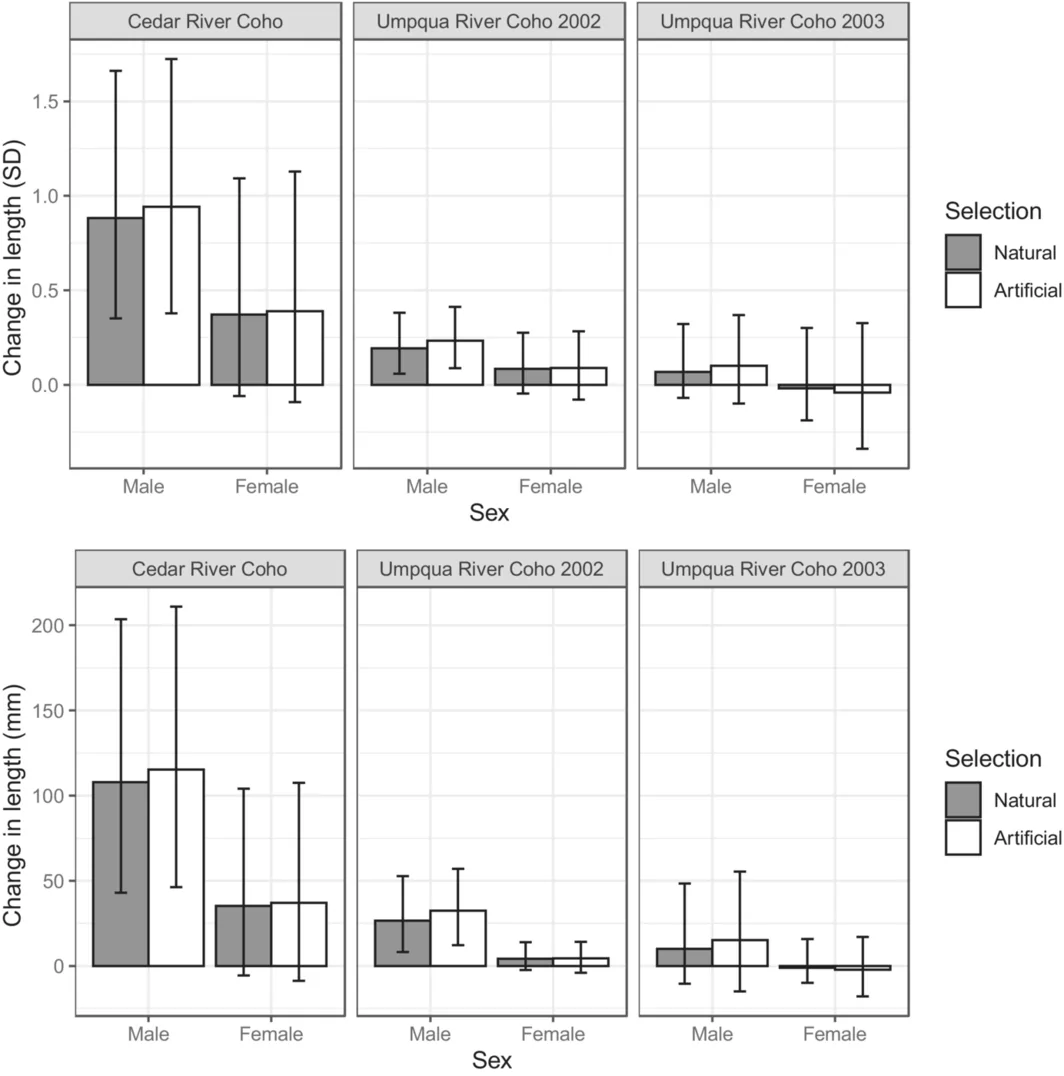
Evolution of length at maturity in a single generation in response to natural selection and artificial selection against jacks, as projected by the breeder's equation.

For Chinook, linear natural selection gradients resulted in a projected evolutionary change of 0.157 (lower–upper uncertainty limits: 0.043–0.346) standard deviations of length at maturity in males and 0.050 (−0.008 to 0.230) standard deviations of length at maturity in females. Using the standard deviation of length for males (117.77 mm) and females (53.53 mm), these results translated to a projected 18.49 mm (5.087 mm–40.806 mm) increase in mean length at maturity for males and a 2.68 mm (−0.435 mm to 12.309 mm) change for females (Figure 2).

For Cedar River Coho, natural selection projected a change of 0.882 (0.351–1.662) standard deviations in males and 0.372 (−0.059 to 1.093) standard deviations in females. Using the standard deviation of length for males (122.39 mm) and females (95.16 mm), these projections translated to a 107.95 mm (42.98 mm–203.42 mm) increase in mean length at maturity for males and a 35.31 mm (−5.57 mm to 104.06 mm) change for females. Artificial selection projected a change of 0.942 (0.378–1.724) standard deviations in males and 0.389 (−0.091 to 1.129) standard deviations in females. These artificial selection projections translated to a 115.27 mm (46.30 mm–210.95 mm) increase in mean length for males and a 37.05 mm (−8.65 mm to 107.43 mm) change for females.

For Umpqua River Coho in 2002, natural selection projected a change in 0.193 (0.059–0.381) standard deviations in males and 0.085 (−0.046 to 0.276) standard deviations in females. Applying these results to the standard deviations of length at maturity for males (138.52 mm) and females (50.38 mm) resulted in a projected 26.69 mm (8.19 mm–52.75 mm) increase in mean length at maturity for males and a 4.30 mm (−2.32 mm to 13.92 mm) change for females. Artificial selection projected a change of 0.234 (0.088–0.412) standard deviations in males and 0.089 (−0.078 to 0.283) standard deviations in females. These projections translated to a 32.46 mm (12.24 mm–57.12 mm) increase in mean length for males and a 4.48 mm (−3.92 mm to 14.26 mm) change for females.

For Umpqua River Coho in 2003, natural selection projected a change of 0.068 (−0.069 to 0.322) standard deviations in males and −0.019 (−0.188 to 0.301) standard deviations in females. Using standard deviations of length for males (150.47 mm) and females (52.67 mm) in this population, natural selection projected a 10.16 mm (−10.33 mm to 48.44 mm) change in mean length at maturity for males and a −1.00 mm (−9.88 mm to 15.86 mm) change in length at maturity for females. Artificial selection projected a change of 0.101 (−0.099 to 0.369) standard deviations in males and −0.041 (−0.339 to 0.326) standard deviations in females, translating to a projected 15.23 mm (−14.94 mm to 55.46 mm) change in mean length for males and a −2.14 mm (−17.83 mm to 17.15 mm) change in mean length for females.

**FIGURE 3:**
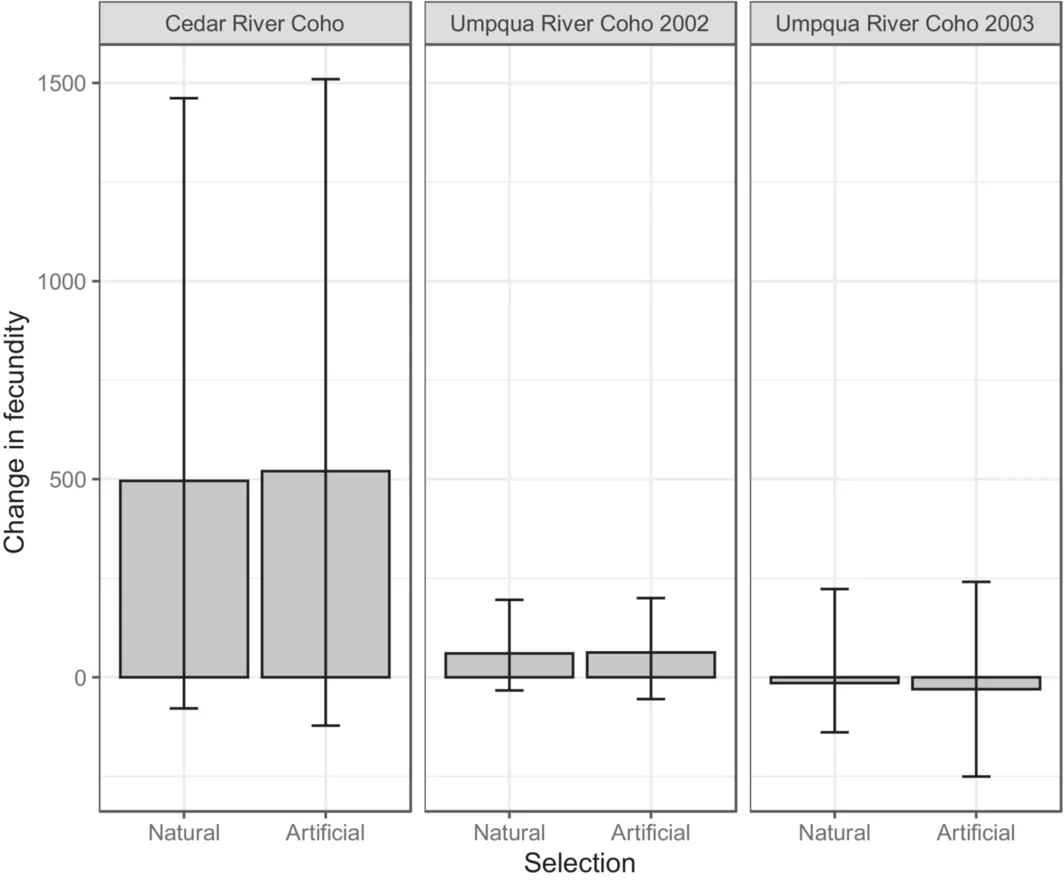
Changes in fecundity due to changes in female length at maturity as projected by the breeder's equation.

Compared to natural selection, artificial selection excluding jacks from hatchery broodstock increased mean fecundity by 25 eggs in the Cedar River Coho population, two eggs in the Umpqua River Coho 2002 population, and decreased mean fecundity by 16 eggs in the Umpqua River Coho 2003 population (Figure 3). The average fecundity of Coho salmon is approximately 2000–4000 eggs (Beacham, 1982); these estimates thus imply a 0.1%–1% change in fecundity due to the exclusion of jacks from broodstock.

## 4 DISCUSSION

We aimed to leverage existing pedigreed datasets from four populations of three Pacific salmon species to understand how selection on male length at maturity could affect the evolution of female length and fecundity. We did so by estimating sex‐specific heritability values of and natural selection on length at maturity in both sexes and simulating artificial selection excluding jacks from hatchery broodstock. We then combined these selection and heritability estimates in the multivariate breeder's equation to estimate the evolution of male and female length at maturity and found that low intersexual heritability estimates for length at maturity mitigated the extent to which selection on males influences evolution in females. Our results shows that while it is possible that intralocus sexual conflict over size at maturity may result in unintended consequences for salmon hatcheries that remove jacks from the breeding pool, we find no evidence that this dynamic plays a large role in the evolution of size in hatchery settings because of very low, near zero, intersexual heritability estimates.

### 4.1 Heritabilities

We have documented positive and significant heritability estimates between parents and their same‐sex progeny across species and populations. By contrast, we also show that heritability estimates between parents and their opposite‐sex progeny were more variable and generally smaller in magnitude than within‐sex heritability estimates, and most did not differ significantly from zero. This was especially true for sire–daughter heritability estimates, which were the lowest in magnitude within each population except in Umpqua River Coho in 2003, in which the sire–daughter heritability estimate was second lowest. The small magnitude of intersexual heritability estimates suggests that female length at maturity may be unlikely to evolve in response to selection on males. While small in magnitude and highly uncertain, both intersexual heritability estimates were negative for Umpqua Coho in 2003. A negative sire–daughter heritability estimate suggests that smaller males have larger daughters. This is consistent with the hypothesis that growth rate is heritable between the sexes, such that faster growing males mature early and small while faster growing females mature at the same age as slower growing females, thus maturing at a larger size at age. Negative intersexual heritability estimates suggest that selection against small males and jacks could reduce female length at maturity, with negative consequences for their fecundity and population growth rates. The possibility of negative intersexual heritability values should be explored further with experimental breeding programs explicitly designed to test for sex‐specific quantitative genetic parameters.

By definition, heritability values cannot be greater than one. However, we estimated a dam‐son heritability of 1.362 ± 0.473 for the Cedar River Coho population. While this estimate is not significantly greater than one, this unrealistically high value may be due to greater relatedness among individuals than expected (inbreeding), to a common environment which makes individuals more similar to one another than expected by shared genes, or to sampling error. We therefore interpret this particular dam‐son heritability estimate as very high or close to, but not in fact greater than, one.

Though we could not estimate heritability values for steelhead due to low sample sizes for each year, the significant negative relationship between the standardized length of dams and sons suggests that larger females have relatively small sons. Again, this could be the result of the significant heritability of growth rate in salmonids generally (Carlson & Seamons, 2008) and steelhead in particular (Hecht et al., 2015), which underlies salmonid maturation schedules such that faster growth leads to earlier maturation (Hecht et al., 2012; Thorpe, 1994), specifically in males. However, further studies should be designed specifically to estimate both intra‐ and intersexual heritability values in steelhead to understand how artificial selection on males may affect female life history, fitness, and population growth rates.

The heritability estimates here are generally higher than others reported for length at maturity in salmonids (median heritability of length at maturity = 0.21, mean = 0.24; Carlson & Seamons, 2008). However, using the data on narrow‐sense heritabilities of length at maturity collected by (Carlson & Seamons, 2008), published heritability estimates of length at maturity in salmonids are higher in males (mean ± SD = 0.31 ± 0.31) than in females (0.18 ± 0.16). Our results also show higher heritability estimates within males compared to females for the Chinook and Cedar River Coho populations, but not the Umpqua Coho population. Very few studies report intersexual heritability estimates for length at maturity. Dickerson et al. (2005) studied a wild population of Pink salmon (*O. gorbuscha*) over 2 years and found that the magnitudes of intersexual and intersexual heritability estimates were similar to each other, counter to our findings that intrasexual heritability estimates are larger in magnitude than intersexual heritability values. However, these estimates were made using raw values of length rather than standardized length and are thus not directly comparable to ours. Another study of pink salmon quantified heritability estimates for length at maturity in sons and daughters separately using mid‐parent length and also found higher heritability estimates in sons (0.45) than daughters (0.34; Funk et al., 2005). Debes et al. (2021) investigated the intersexual genetic correlation between early male maturity and female length, though not length at maturity, in Atlantic salmon over 12 months and found that it decreased over time and that its confidence interval overlapped with zero at 12 months, also suggesting agreement with our results.

There are multiple proximate mechanisms that may result in low intersexual heritability values, and these may vary among populations and species. Sex‐specific dominance at loci of large effect have been found in Atlantic salmon (Ayllon et al., 2015; Barson et al., 2015) and Steelhead trout (Pearse et al., 2019). In Chinook salmon, genes associated with age at maturity have been found to be in linkage disequilibrium with the sex determining gene *sdY* in males, whereas the genetic architecture of age at maturity in females is much more polygenic (McKinney et al., 2020). Both of these mechanisms may resolve potential sexual conflict over age and size at maturity.

### 4.2 Selection

Linear selection gradients were positive for both sexes in all populations and years, suggesting directional natural selection favoring longer fish. The strength of directional selection in males was more than twice that of females in 3 of 4 populations and years, indicating that males have more to gain than females from being large. This is likely because males can mate more than females due to the low cost of sperm compared to eggs (Bateman, 1948), and larger males may gain access to more females (Berejikian et al., 2010; Quinn, 2018), while even small females will usually attain some reproductive success (Shuster & Wade, 2003). Combined with the negative intersexual heritability estimates in the Umpqua Coho population in 2003 and the negative intersexual parent–offspring regressions in the steelhead population, the fact that selection favors larger males and females in both these populations indicates the potential for intralocus sexual conflict in these populations and a constraint on adaptive evolution (Bonduriansky & Chenoweth, 2009; Rice & Chippindale, 2001). This is borne out by the fact that the breeder's equation predicts a reduction in mean female length due to natural selection in Umpqua Coho, as discussed below.

Most quadratic selection gradients were small in magnitude and did not differ significantly from zero (Table 2). There were three exceptions to this lack of quadratic selection: Females experienced significant negative quadratic selection in the Cedar River Coho population and in the Umpqua River Coho population in 2003, and males experienced significant positive quadratic selection in the Umpqua River Coho population in 2002. Negative quadratic selection is likely due to the possibility of stranding and predation in shallow spawning streams (Quinn et al., 2001). Positive quadratic selection in males is likely due to the presence of alternative reproductive tactics: large and small males gain reproductive success using different strategies, while intermediate‐sized males cannot use either strategy effectively (Gross, 1985).

In all populations and years, artificial selection on males estimated by removing jacks from broodstocks resulted in linear selection gradients that were 20%–40% greater than natural linear selection gradients. Though these selection gradients are hypothetical, they mirror the complete exclusion of jacks in salmon hatcheries and are thus useful for understanding how excluding jacks from broodstocks affects selection on female length.

### 4.3 Breeder's equation projections

The validity of predictions from the multivariate breeder's equation depend on the degree to which all relevant traits (and corresponding genetic variances and covariances) have been measured (Lande & Arnold, 1983). Nowhere is this task more difficult than in studies of wild populations (Morrissey et al., 2010). Additionally, the breeder's equation and other quantitative genetic methods are only useful for short‐term evolutionary predictions unless evolution of the G matrix is explicitly taken into account (Lande, 1979; Steppan et al., 2002). Nevertheless, the multivariate breeder's equation remains a valuable heuristic tool for exploring how artificial selection on male length could alter evolution in females given estimated intersexual heritability values between male and female length at maturity and is used here for illustrative purposes.

In all populations and years, artificial selection excluding jacks resulted in a greater projected change in mean length at maturity than did natural selection in both sexes. Because intrasexual heritability estimates were greater in magnitude than intersexual heritability estimates, the difference in projected evolutionary change (in units of standard deviations) between natural and artificial selection was greater in males than in females in all populations and years. However, artificial selection against jacks did alter projections of the evolution of female length at maturity, and in the Umpqua River Coho population in 2003, both natural and artificial selection resulted in projected decreases in female length at maturity due to negative sire–daughter heritability estimates. This demonstrates that selection on male alternative reproductive tactics can affect evolution in females. This result is consistent with theory (Gamble & Calsbeek, 2023) and empirical studies (Bielak et al., 2014; Sinervo & Zamudio, 2001), and has important implications for conservation and hatchery practices. Specifically, hatchery practices that limit or fully exclude jacks from broodstock may result in unintentional and undesirable outcomes, such as reduced female size and fecundity. Indeed, given the history of the breeder's equation as a tool developed and used in the livestock and agriculture industries (Hill, 2014), it is paradoxical that current hatchery practices do not consider the fact that negative intersexual genetic correlations can change the predictions of the breeders equation such that selection on a trait of interest in one sex may move that trait in the other direction in the other sex.

We projected that excluding jacks from hatchery broodstock could alter female fecundity by about 1%, and he lower bounds of our fecundity estimate results suggest the possibility of declining fecundity due to selection against jacks. While small, this change could be relevant to conservation policy, as declines in female length and fecundity are currently of great concern in some salmon populations (Lewis et al., 2015; Ohlberger et al., 2020; Oke et al., 2020). While size‐selective harvest, competition for food in the ocean (both among species and between hatchery‐ and wild‐origin fish), and climate change are all often considered as potential mechanisms underlying this trend, artificial selection against jacks in hatcheries has not been considered.

Estimates of selection and inter‐ and intrasexual heritability values could be affected if size‐biased sampling methods resulted in an under‐representation of jacks in the datasets. However, the sampling methods used to collect each dataset used in this analysis make it unlikely that sampling was biased against jacks, as in each case all fish returning to spawn were captured and sampled at weirs or dams (Anderson et al., 2010; Ford et al., 2012; Seamons et al., 2007; Thériault et al., 2011). However, because these methods rely on migration, they also ensure that mature male parr were not sampled. We must thus restrict our conclusions to the ways in which jacks might affect the fitness of their daughters. Future studies should include mature male parr, as they may represent significant portions of spawning males in certain populations and species (Fleming, 1998; Myers, 1984; Quinn, 2018).

Additional shortcomings of our approach stem from the fact that we use datasets collected at different times, by different groups of people, using different methods. Differences in parentage analysis methods may render comparisons among populations less useful if parentage analysis was more successful or accurate in some populations than others. This would affect both heritability estimates and selection analyses, because identifying parent–offspring relationships is the basis for both heritability and fitness estimates. Additionally, each dataset defines natural‐origin and hatchery‐origin individuals somewhat differently. In all but the steelhead population, natural‐origin fish may have significant hatchery ancestry. This inconsistency may impact the interpretation of our artificial selection results, since these fish may themselves be the product of artificial selection. Nevertheless, we feel the value of the concepts and analyses presented here overcomes these unavoidable drawbacks associated with using existing datasets.

## 5 CONCLUSIONS

We have shown that between‐sex heritability estimates of length at maturity are generally much lower than within‐sex heritability estimates, suggesting that selection on male length will have limited effects on the evolution of female length and fecundity. However, we have also demonstrated that artificial selection against jacks in hatcheries may alter evolutionary trajectories compared with natural selection, and the wide uncertainty around our intersexual heritability estimates often include negative values. Though we do not have evidence for negative intersexual heritability values in these populations, we have demonstrated that artificial selection against small males has the potential to reduce mean female length, and thus fecundity, if intersexual heritability estimates are in fact negative. Because fecundity influences population growth rate, this insight is crucial for developing evolutionarily informed conservation and hatchery policies.

## CONFLICT OF INTEREST STATEMENT

The authors declare no conflicts of interest.

## Supporting information


Appendix S1.

## Data Availability

All data and R code for this study will be made publicly available upon manuscript acceptance. Data for the Umpqua Coho population are publicly available at the following URL: http://ir.library.oregonstate.edu/concern/datasets/8336h610p.
