# Supplementary material for: Sex‐specific heritabilities for length at maturity among Pacific salmonids and their consequences for evolution in response to artificial selection
Source: Evol Appl. 2023 Jul 11;16(8):1458–71. doi: 10.1111/eva.13579 (PMC10445087; doi:10.1111/eva.13579)
Supplement: Supplementary file 1 — Appendix S1. [file EVA-16-1458-s001.docx]

**Supplemental Material**

**Supplemental Methods:**

**Preliminary analysis suggesting potential for significant sex-specific heritability:**

To test whether the heritability of this standardized length differed based on the sex of the offspring and the sex of the parent, we used linear models for each population and year relating offspring standardized length to dam standardized length, sire standardized length, offspring sex, and all two-way interactions:

$OffspringStdLength = {}_{0}+{}_{1}\left( \mathrm{Dam}\mathrm{Std}\mathrm{Length} \right)+ {}_{2}\left( \mathrm{Sire}\mathrm{Std}\mathrm{Length} \right)+ {}_{3}\left( \mathrm{OffspringSex} \right)+ {}_{4}\left( DamStdLength*SireStdLength \right)+ {}_{5}\left( \mathrm{Dam}\mathrm{Std}Length*OffspringSex \right)+ {}_{6}(Sire\mathrm{Std}Length*OffspringSex)$ + ε

Significant interactions between parent length and offspring sex would indicate that dam- or sire-specific heritabilities differed between sons and daughters, while a significant interaction between sire and dam length would indicate different parent-offspring relationships depending on parent sex. Results are reported in Table S1.

**Supplemental Tables:**

**Table S1.** Results from linear models for each population relating offspring standardized length to dam standardized length, sire standardized length, offspring sex, and all two-way interactions on offspring length.

| **Parameter** | **Coefficient** | **DF** | ***F* value** | ***p* value** |
| --- | --- | --- | --- | --- |
| Chinook (Wenatchee River, WA) – 2004 | | | | |
| Intercept | 0.83 | 1 | 1.042 | 0.3 |
| Dam Std Length | 1.89 | 1 | 2.366 | 0.1 |
| Sire Std Length | 1.19 | 1 | 1.486 | 0.2 |
| Offspring Sex | 0.08 | 1 | 0.104 | 0.7 |
| Dam Std Length * Sire Std Length | 0.12 | 1 | 0.155 | 0.7 |
| Dam Std Length * Offspring Sex | 0.06 | 1 | 0.070 | 0.8 |
| Sire Std Length * Offspring Sex | 4.96 | 1 | 6.211 | 0.01 |
| Coho (Cedar River, WA) – 2005 | | | | |
| Intercept | 1.30 | 1 | 1.490 | 0.2 |
| Dam Std Length | 11.17 | 1 | 12.829 | 0.0004 |
| Sire Std Length | 0.76 | 1 | 0.875 | 0.4 |
| Offspring Sex | 1.37 | 1 | 1.572 | 0.2 |
| Dam Std Length * Sire Std Length | 1.10 | 1 | 1.258 | 0.3 |
| Dam Std Length * Offspring Sex | 2.03 | 1 | 2.336 | 0.1 |
| Sire Std Length * Offspring Sex | 0.94 | 1 | 1.075 | 0.3 |
| Coho (Umpqua River, OR) – 2002 | | | | |
| Intercept | 0.14 | 1 | 0.148 | 0.7 |
| Dam Std Length | 14.8 | 1 | 15.752 | < 0.0001 |
| Sire Std Length | 0.28 | 1 | 0.293 | 0.6 |
| Offspring Sex | 0.04 | 1 | 0.046 | 0.8 |
| Dam Std Length * Sire Std Length | 0.05 | 1 | 0.053 | 0.8 |
| Dam Std Length * Offspring Sex | 1.82 | 1 | 1.937 | 0.2 |
| Sire Std Length * Offspring Sex | 5.19 | 1 | 5.519 | 0.02 |
| Coho (Umpqua River, OR) – 2003 | | | | |
| Intercept | 0.08 | 1 | 0.083 | 0.8 |
| Dam Std Length | 2.81 | 1 | 2.838 | 0.09 |
| Sire Std Length | 0.16 | 1 | 0.162 | 0.7 |
| Offspring Sex | 0.10 | 1 | 0.097 | 0.8 |
| Dam Std Length * Sire Std Length | 0.58 | 1 | 0.580 | 0.4 |
| Dam Std Length * Offspring Sex | 4.71 | 1 | 4.755 | 0.03 |
| Sire Std Length * Offspring Sex | 1.15 | 1 | 1.161 | 0.3 |
| Steelhead (Snow Creek, WA) – 1982-2000 | | | | |
| Intercept | 1.48 | 1 | 2.6009 | 0.1 |
| Dam Std Length | 5.77 | 1 | 10.1539 | 0.002 |
| Sire Std Length | 0.03 | 1 | 0.0580 | 0.8 |
| Offspring Sex | 1.07 | 1 | 1.8886 | 0.2 |
| Dam Std Length * Sire Std Length | 0.12 | 1 | 0.2015 | 0.7 |
| Dam Std Length * Offspring Sex | 4.08 | 1 | 7.1732 | 0.008 |
| Sire Std Length * Offspring Sex | 1.90 | 1 | 3.3342 | 0.07 |

**Table S2.** Coefficients and statistical parameters of single parent-offspring regressions for standardized length at maturity for multiple species and populations of Pacific salmon. *P* values associated with *t* statistics refer to tests of the hypothesis that regression slopes are different from 0. Heritability estimates are not reported for steelhead as they cannot be estimated across multiple generations. Bootstrapped mean and 95% CI heritability estimates are reported for Chinook and Coho populations; bootstrapped mean and 95% CI slope estimates are reported for steelhead.

| **Model (Offspring~**  **Parent)** | **Slope Estimate ± SE** | ***t*** | ***p*** | ***h^2^* ± 95%CI** | **Bootstrapped *h^2^* (95%CI)** |
| --- | --- | --- | --- | --- | --- |
| Chinook (Wenatchee River, WA) – 2004 | | | | | |
| Daughter~Dam | 0.132 ± 0.113 | 1.17 | 0.2 | 0.264 ± 0.443 | 0.264 (-0.230, 0.738) |
| Son~Dam | 0.221 ± 0.088 | 2.52 | 0.01 | 0.441 ± 0.343 | 0.448 (0.114, 0.781) |
| Daughter~Sire | 0.120 ± 0.126 | 0.96 | 0.3 | 0.240 ± 0.492 | 0.269 (-0.318, 0.915) |
| Son~Sire | 0.468 ± 0.082 | 5.7 | < 0.0001 | 0.936 ± 0.322 | 0.936 (0.526, 1.241) |
| Coho (Cedar River, WA) – 2005 | | | | | |
| Daughter~Dam | 0.325 ± 0.164 | 2 | 0.055 | 0.651 ± 0.642 | 0.627 (-0.195, 1.210) |
| Son~Dam | 0.446 ± 0.092 | 4.82 | < 0.0001 | 1.362 ± 0.473 | 1.365 (0.891, 1.756) |
| Daughter~Sire | 0.123 ± 0.147 | 0.83 | 0.4 | 0.245 ± 0.578 | 0.220 (-0.448, 1.017) |
| Son~Sire | 0.427 ± 0.147 | 2.91 | 0.006 | 0.855 ± 0.576 | 0.849 (0.237, 1.366) |
| Coho (Umpqua River, OR) – 2002 | | | | | |
| Daughter~Dam | 0.265 ± 0.077 | 3.43 | 0.0007 | 0.531 ± 0.302 | 0.526 (0.255, 0.805) |
| Son~Dam | 0.129 ± 0.072 | 1.89 | 0.07 | 0.258 ± 0.282 | 0.259 (0.041, 0.601) |
| Daughter~Sire | 0.022 ± 0.079 | 0.27 | 0.8 | 0.044 ± 0.311 | 0.053 (-0.201, 0.292) |
| Son~Sire | 0.263 ± 0.072 | 3.67 | 0.0003 | 0.527 ± 0.281 | 0.524 (0.234, 0.903) |
| Coho (Umpqua River, OR) – 2003 | | | | | |
| Daughter~Dam | 0.144 ± 0.129 | 1.12 | 0.3 | 0.289 ± 0.507 | 0.298 (-0.235, 0.813) |
| Son~Dam | -0.072 ± 0.097 | -0.74 | 0.5 | -0.148 ± 0.382 | -0.143 (-0.547, 0.266) |
| Daughter~Sire | -0.075 ± 0.125 | -0.60 | 0.6 | -0.156 ± 0.492 | -0.163 (-0.309, 0.470) |
| Son~Sire | 0.122 ± 0.101 | 121 | 0.2 | 0.244 ± 0.395 | 0.239 (-0.160, 0.718) |
| Steelhead (Snow Creek, WA) – 1982-2000 | | | | | **Bootstrapped Slope (95%CI)** |
| Daughter~Dam | 0.232 ± 0.072 | 3.21 | 0.002 |  | 0.280 (0.115, 0.481) |
| Son~Dam | -0.181 ± 0.081 | -2.23 | 0.03 |  | -0.184 (-0.413, -0.037) |
| Daughter~Sire | -0.008 ± 0.127 | -0.06 | 0.9 |  | -0.001 (-0.220, 0.223) |
| Son~Sire | 0.294 ± 0.121 | 2.42 | 0.02 |  | 0.289 (0.010, 0.618) |

**Supplemental Figures:**

**Figure S1.** Histogram of male length used to identify jacks in the Cedar River Coho population.

**Figure S2.** Intersexual heritability of length at maturity for spring-run Chinook salmon from the Wenatchee River, WA. Lengths are standardized (mean = 0, SD = 1) within brood year and sex. Regression lines are bold where the slope differs significantly from 0. Dashed lines show 95% CI. Size of the data points is proportional to the number of offspring comprising the family mean.

**Figure S3.** Intersexual heritability of length at maturity for Coho salmon from Cedar River, WA. Lengths are standardized (mean = 0, SD = 1) within brood year and sex. Regression lines are bold where the slope differs significantly from 0. Dashed lines show 95% CI. Size of the data points is proportional to the number of offspring comprising the family mean.

**Figure S4.** Intersexual heritability of length at maturity for Coho salmon from the Umpqua River, OR. Lengths are standardized (mean = 0, SD = 1) within brood year and sex. Regression lines are bold where the slope differs significantly from 0. Dashed lines show 95% CI. Size of the data points is proportional to the number of offspring comprising the family mean.

**Figure S5.** Intersexual heritability of length at maturity for steelhead from Snow Creek, WA. Lengths are standardized (mean = 0, SD = 1) within brood year and sex. Regression lines are bold where the slope differs significantly from 0. Dashed lines show 95% CI. Size of the data points is proportional to the number of offspring comprising the family mean.

**Figure S1**. Histogram of male length used to identify jacks in the Cedar River Coho population.

**Figure S2.** Intersexual heritability of length at maturity for spring-run Chinook salmon from the Wenatchee River, WA. Lengths are standardized (mean = 0, SD = 1) within brood year and sex. Regression lines are bold where the slope differs significantly from 0. Dashed lines show 95% CI. Size of the data points is proportional to the number of offspring comprising the family mean.

**Figure S3.** Intersexual heritability of length at maturity for Coho salmon from Cedar River, WA. Lengths are standardized (mean = 0, SD = 1) within brood year and sex. Regression lines are bold where the slope differs significantly from 0. Dashed lines show 95% CI. Size of the data points is proportional to the number of offspring comprising the family mean.

**Figure S4**. Intersexual heritability of length at maturity for Coho salmon from the Umpqua River, OR. Lengths are standardized (mean = 0, SD = 1) within brood year and sex. Regression lines are bold where the slope differs significantly from 0. Dashed lines show 95% CI. Size of the data points is proportional to the number of offspring comprising the family mean.

**Figure S5.** Intersexual parent-offspring regressions of length at maturity for steelhead from Snow Creek, WA. Lengths are standardized (mean = 0, SD = 1) within brood year and sex. Regression lines are bold where the slope differs significantly from 0. Dashed lines show 95% CI. Size of the data points is proportional to the number of offspring comprising the family mean.
